# Lipid Nanocapsule-Based Gels for Enhancement of Transdermal Delivery of Ketorolac Tromethamine

**DOI:** 10.1155/2011/571272

**Published:** 2011-11-17

**Authors:** Jaleh Varshosaz, Valiollah Hajhashemi, Sindokht Soltanzadeh

**Affiliations:** ^1^Department of Pharmaceutics, Faculty of Pharmacy and Isfahan Pharmaceutical Sciences Research Center, Isfahan University of Medical Sciences, Isfahan 81746-73461, Iran; ^2^Department of Pharmacology, Faculty of Pharmacy and Isfahan Pharmaceutical Sciences Research Center, Isfahan University of Medical Sciences, Isfahan 81746-73461, Iran

## Abstract

Previous reports show ineffective transdermal delivery of ketorolac by nanostructured lipid carriers (NLCs). The aim of the present work was enhancement of transdermal delivery of ketorolac by another colloidal carriers, lipid nanocapsules (LNCs). LNCs were prepared by emulsification with phase transition method and mixed in a Carbomer 934P gel base with oleic acid or propylene glycol as penetration enhancers. Permeation studies were performed by Franz diffusion cell using excised rat abdominal skin. Aerosil-induced rat paw edema model was used to investigate the *in vivo* performance. LNCs containing polyethylene glycol hydroxyl stearate, lecithin in Labrafac as the oily phase, and dilution of the primary emulsion with 3.5-fold volume of cold water produced the optimized nanoparticles. The 1% Carbomer gel base containing 10% oleic acid loaded with nanoparticles enhanced and prolonged the anti-inflammatory effects of this drug to more than 12 h in Aerosil-induced rat paw edema model.

## 1. Introduction

Lipid nanocapsules (LNCs) are a new generation of biomimetic nanovectors composed of an oily core of medium-chain triglycerides of capric and caprylic acids known under the commercial name of Labrafac that is surrounded by a shell composed of lecithin and a pegylated surfactant called Solutol HS 15. Solutol is a mixture of free PEG 660 and PEG 660 hydroxystearate and oriented towards the water phase. Lecithin is composed of 69% phosphatidylcholine soya bean and is generally used in small proportions to significantly increase LNC stability [1, 2]. Their structure mimics lipoproteins [3, 4] while have a hybrid structure between polymer nanocapsules and liposomes. LNCs present a great physical stability up to 18 months with size ranges from 20 to 100 nm. They are prepared by a phase inversion of an oil/water emulsion due to thermal manipulation and in the absence of organic solvents with good monodispersion [5]. The aqueous phase consists of MilliQ water plus sodium chloride salt, which helps to decrease the phase-inversion temperature (PIT) [5, 6].

Preparation of LNCs involves two steps. In the first step all mixed components are heated from room temperature up to T2 temperature, above the PIT, to obtain a W/O emulsion. Then the temperature is dropped to T1 below the PIT, by a cooling process that leads to the formation of an O/W emulsion. After several temperature cycles between T2 and T1 the temperature is set 1–3°C lower from the beginning of the O/W emulsion before dilution. At the second step by a sudden dilution with cold water added to the mixture an irreversible shock causes to break the microemulsion system, and stable nanocapsules are formed [7]. Three temperature cycles of heating and cooling at the rate of 4°C/min are usually applied between 85 and 60°C [5, 8].

They have been used from different routes of administration including oral [9, 10], parenteral, and transdermal routes [11–13]. Improved bioavailability, increased drug targeting, achieving controlled drug release [14–16], increasing the stability of the entrapped drugs, low biotoxicity, and good biocompatibility are some advantages reported for LNCs [17]. 

The gastrointestinal side effects of nonsteroidal anti-inflammatory drugs (NSAIDs) have limited their widely oral use as analgesics in the treatment of local inflammation. This has prompted researchers to investigate the feasibility of alternative dermal and/or transdermal drug delivery systems. Ketorolac is a pyrrolizine carboxylic acid derivative of NSAIDs with potent analgesic and moderate anti-inflammatory activity, a relatively favorable therapeutic agent for the management of moderate to severe pain [18]. Ketorolac tromethamine is administered intramuscularly and orally in divided multiple doses for short-term management of postoperative pain. Its oral bioavailability is 90% with a very low first pass metabolism. However, the drug is reported to cause severe gastrointestinal side effects such as gastrointestinal bleeding, perforation, peptic ulceration, and acute renal failure [19]. Because of the short half-life (4 to 6 h) of ketorolac, frequent dosing is required to alleviate pain. To avoid intramuscular injection and frequent dosing regimens, dermal and transdermal delivery of ketorolac is an attractive alternative. Additionally, high analgesic activity and low molecular weight of ketorolac make it a good candidate for transdermal delivery. Several transdermal delivery strategies such as use of permeation enhancers [20], proniosomes [21], its prodrugs [22], iontophoresis [23], ultrasound [24], cyclodextrins and liposomes [25], and nanostructured lipid carriers (NLCs) [26] have been developed so far. NLCs are mixtures of solid and liquid lipids (oils) which provide greater solubility for drugs than solid lipids. These nanostructures of ketorolac were ineffective in increasing the drug percutaneous absorption due to the high degree of mutual interaction between the drug and carrier lipid matrix. For this reason we propose another colloidal lipid nanocarrier, that is, LNCs for transdermal delivery of ketorolac due to their high content of hydrophilic surfactants which may improve the problem of previous nanoparticles of this drug and reduce high degree of interactions between the drug and nanoparticles. The LNCs are prepared by an emulsification-phase conversion process with 10–40% or more of surfactants and contain no organic solvent.

## 2. Materials and Methods

### 2.1. Materials

Ketorolac tromethamine (MSN Laboratory, India), Labrafac (capric and caprylic acid triglyceride) and polyethylene glycol hydroxyl stearate (Solutol HS15) were kindly provided by BASF (The Chemical Company, Ludwigshafen, Germany), Carbomer P934 (BF Goodrich, US), soy lecithin S100 (Lipoid, Germany), Aerosil (Fluka, US), triethanolamine (Sigma, US), oleic acid, propylene glycol, Tween 20 and all other reagents were from Merck, Chemical Company (Germany). Ketamine 10% vial was from (Alfasan, Netherland).

### 2.2. Preparation and Optimization of LNCs Using Taguchi Design

Table 1 displays the four control factors that were selected in the optimization study. A standard orthogonal array L_9_ [27] was used to examine this four-factor system. L and subscript 9 denote the Latin square and the number of the experimental runs, respectively. A run involved the corresponding combination of levels to which the factors in the experiment were set. All studied factors had three levels. All experiments were performed in triplicate. 

Four studied responses included particle size, zeta potential, loading efficiency, and drug release efficiency percent until 65 min (RE_65_%). The experimental results were then analyzed by the Design Expert software (version 7, USA) to extract independently the main effects of these factors, followed by the analysis of variance (ANOVA) to determine which factors were statistically significant. Identifying controlling factors and qualifying the magnitude of effects, as well as identifying the statistically significant effects, were emphasized. The optimum conditions were determined by the Taguchi's optimization method [28] to yield a heightened performance with the lowest possible effect of the noise factor. 

To prepare the LNCs 400 mg drug was dissolved in 2.73 mL of aqueous phase containing 1.75% NaCl (according to the aqueous phase) and different amounts of polyethylene glycol hydroxyl stearate as the surfactant (according to Table 1). The oily phase was Labrafac which contained lecithin as the stabilizing agent. The amount of each variable is shown in Table 1. The two phases were added to each other on a magnetic stirrer, and the mixture temperature was raised from room temperature to 85°C gradually during 15 min. Then it was cooled to 25°C. Three temperature cycles (85–60–85–60–85°C) were applied to reach the inversion process. The temperature of the mixture before dilution was set 57°C, in the o/w emulsion. Step II was an irreversible shock induced by dilution (1.2–3.5 times) with cold de-ionised water (0°C) added to the mixture maintained at the previously defined temperature. This fast-cooling dilution process led to the formation of stable nanocapsules. Afterwards slow magnetic stirring for 5 min was applied to the suspension [7].

### 2.3. Particle Size and Zeta Potential of the LNCs

Size and zeta potential of all drug-loaded LNC samples were measured by photon correlation spectroscopy (PCS, Zetasizer 3000, Malvern, UK). All the samples were diluted one to ten ratio with deionized water to get optimum 50–200 kilo counts per second (Kcps) for measurements. Intensity Z-Average particle size, polydispersity index, and zeta potential were measured.

### 2.4. Morphology Study

Morphology of the LNCs was characterized by scanning electron microscopy (SEM). The nanoparticles were mounted on aluminum stubs, sputter coated with a thin layer of Au/Pd, and examined using an SEM (Seron Technology 2008, Korea).

### 2.5. Drug Loading Efficiency in LNCs

Entrapment efficiency percent (EE%) was determined by measuring the concentration of unentrapped free drug in aqueous medium [29]. The aqueous medium was separated by centrifugation (Sigma 3K30, Germany). About 0.5 mL of the LNC dispersion was placed in the eppendorf Amicon Ultra centrifugal filters with cut-off 10 KDa and centrifuged at 15000 rpm for 10 min. The encapsulated drug in nanoparticles was separated, and the amount of free ketorolac in the aqueous phase was estimated by UV spectroscopy method at *λ*_max⁡_ = 319.3 nm. The EE% and loading percentage were calculated using:

(1) $$\begin{array}{cc} & \text{EE}\;\left(\%\right)\\ & =\left(\frac{\text{Analyzes}\;\text{weight}\;\text{of}\;\text{drug}\;\text{in}\;\text{LNC}}{\text{Theoretical}\;\text{weight}\;\text{of}\;\text{drug}\;\text{loaded}\;\text{in}\;\text{system}}\right)\times 100,\\ & \text{Loading}\;\text{\%}\\ & =\left(\frac{\text{Analyzed}\;\text{weight}\;\text{of}\;\text{drug}\;\text{in}\;\text{LNC}}{\text{Analyzed}\;\text{weight}\;\text{of}\;\text{LNC}}\right)\times 100. \end{array}$$

### 2.6. Drug Release Studies from LNCs

To determine the release rate of ketorolac from nanoparticles 1 mL of aqueous dispersion of each formulation was added to the dialysis bags with molecular weight cutoff of 12400 Da, and the sealed bags were placed in the glass test tube in 25 mL of the phosphate buffer solution (PBS) 0.1 M (pH 7.4) to provide sink conditions with agitation of 200 rpm on magnetic stirrer. Samples were withdrawn at 15 min time intervals up to 65 min and replaced with fresh PBS maintained at the same temperature. The content of ketorolac in the samples was determined spectrophotometrically at *λ*_max⁡_ = 323 nm.

### 2.7. Preparation and Optimization of LNC-Based Gels Using Factorial Design

Three different variables each in 2 levels were evaluated for preparation of the gel bases (Table 2). The gel formulations were prepared using a 2-level factorial design. Carbomer was dispersed in water using an overhead stirrer at a speed of 600 rpm for 3 hr. Carbomer gels were diluted to final concentration of 0.5–1% with the optimized formulation of LNCs and then neutralized using 0.5 w/w% triethanolamine. Then one of the absorption enhancers (oleic acid or propylene glycol) was added at different concentrations (Table 2).

### 2.8. Skin Permeation through Excised Hairless Rat

In vitro permeation of ketorolac from various gel formulations was evaluated using full thickness abdominal skin excised from adult Wistar rats weighing 150–180 g. The visceral side of the freshly excised skin was cleaned free of any adhering subcutaneous tissue. The hair on the epidermal surface of the skin was cut, and the skin was hydrated for 24 h in PBS (pH 7.4). The skin samples were mounted on Franz diffusion cells with a diameter of 2.6 cm and a receptor volume of 28 mL such that the dermal side of the skin was exposed to the receptor fluid and the stratum corneum remained in contact with the donor compartment. PBS (pH 7.4) was filled in the receptor compartment and stirred continuously with the help of a magnetic stirrer. The receptor medium was water jacketed at 37°C. On the epidermal side of the skin, 1 g of the gel was spread evenly. Two mL samples were withdrawn from receptor medium and replaced with fresh medium at 0.5, 1, 2, 4, 16, and 17 h. Samples were analyzed spectrophotometrically for the content of ketorolac at 323 nm. Blank formulations (without drug) were used as a reference for the determination of ketorolac to negate any possible interference from the skin components or formulation components. Cumulative amount of drug (*Q*) permeated through skin was plotted as a function of time (*t*). The drug concentration in the donor cell (*C*_*d*_) and its surface area (*S*) were used for calculation of the permeability (*P*):

(2) $$Q=PSC_{d}t.$$

Flux (*J*_*s*_) was calculated from (3) in which (*dQ*/*dt*) is the amount of drug flowing through a unit cross-section (*S*) of the skin in unit time (*t*)

(3) $$J_{S}=\frac{1}{S}\frac{dQ}{dt}.$$

To obtain the diffusion coefficient (*D*) of the drug through the skin (4) was used:

(4) $$t_{L}=\frac{h^{2}}{6D},$$

In which (*t*_*L*_) is the lag time of drug permeation and (*h*) is the thickness of the rat skin. Finally the partition coefficient (*K*_*m*_) of drug between skin and vehicle was obtained from:

(5) $$K_{m}=\frac{P\cdot D}{h}.$$

All the experiments were performed in triplicate. After optimization of the gel formulation according to the highest skin permeability, the optimized gel was applied for *in vivo* studies in alleviating the Aerosil-induced paw edema in rat.

### 2.9. *In Vivo* Studies

#### 2.9.1. Animals

36 male albino Wistar rats with body weight of 150–180 g (70–90 days aged) were selected for all the experiments. Animals were kept in the animal house at 23–30°C and 45–55% relative humidity. The Isfahan University of Medical Sciences ethical committee approved all animal experiments in the present study.

#### 2.9.2. Aerosil-Induced Paw Edema in Rats

Male Wistar rats were studied into 6 groups of six rats, each group receiving a different topical treatment. 0.1 mL of 2.5% Aerosil suspension in distilled water was injected in the right hind foot of each rat. Immediately after injection of Aerosil the rats of the test groups were administered the developed optimized LNC-based gels containing 0.5 or 2% ketorolac, the 2 standard groups were treated with the traditional gels of 0.5 or 2% free ketorolac in the same gel base as LNCs, the control group received no treatment, and another group received the blank vehicle. 

Measurement of the foot volume was performed by the displacement technique using plethysmograph (Ugo Basile, Italy) immediately before and 2, 4, 8, 12, and 24 h after the injection of Aerosil. Edema inhibition rate (*I*) after different treatments was calculated using:

(6) $$\begin{array}{cc} E & =\frac{V_{t}-V_{o}}{V_{o}},\\ I\% & =\frac{E_{c}-E_{t}}{E_{c}}\times 100, \end{array}$$

where *V*_*o*_ is the mean paw volume before Aerosil injection, *V*_*t*_ is the mean paw volume after Aerosil injection, *E*_*c*_ is the edema rate of the control group, and *E*_*t*_ is the edema rate of the treated group [30].

### 2.10. Statistical Analysis

SPSS software version 11.5 was used for all statistical analysis. One-way analysis of variance (ANOVA) followed by a Tukey's post hoc test was used for comparison between cumulative percentage of drug released at the end of each release test. 

*In vivo* data were expressed as mean ± SD. Differences between mean values were analyzed using one-way analysis of variance (ANOVA) followed by a Dunnett's post hoc. A significant level of *P* < 0.05 denoted significance in all cases.

## 3. Results and Discussion

Different formulations of ketorolac LNCs were prepared according to Table 3 and were characterized for their physical properties including particle size, surface charge (zeta potential), drug loading efficiency percent, and release efficiency until 65 min of release test (RE_65_%). The results are shown in Table 3. Optimization was performed to obtain the optimal points regarding the constraints in which the particle size was in its minimum level, the absolute value of zeta potential in its maximum level, while release efficiency percent and loading efficiency percent were in their range levels (Table 3).

**Figure 1:**
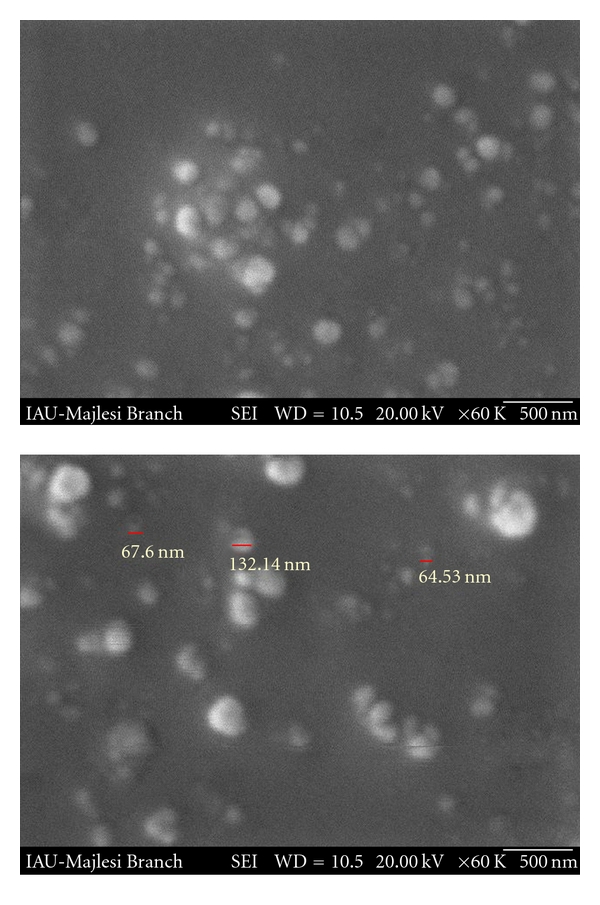
SEM micrographs of optimized formulation of Ketorolac loaded lipid nanocapsules (S_20_O_25_W_3.5_L_3.25_).

The optimized formulation of LNCs was predicted by Design Expert software to contain 20% polyethylene glycol hydroxyl stearate (coded as level I), 25% Labrafac, 3.25% lecithin, and diluting cool water 3.5-fold of the volume of the primary emulsion (all coded as level III in Table 1). The optimized formulation of LNCs was prepared and characterized for their physical properties (Table 3). The nanoparticulate nature of the LNC dispersion was confirmed by SEM studies (Figure 1), otherwise some aggregation of nanoparticles is obvious. The morphology of this optimized LNC formulation is seen in Figure 1.

**Figure 2:**
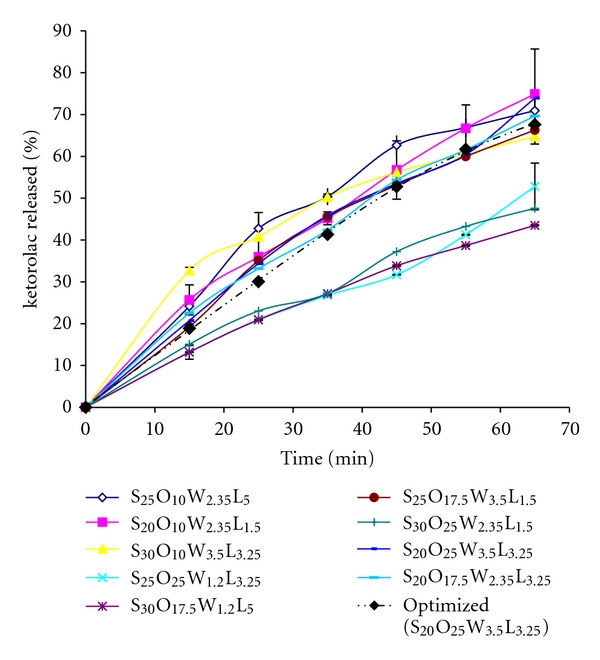
Ketorolac release profile from lipid nanocapsules with different formulations.

Drug release profiles through LNC formulations are compared with the optimized formulation in Figure 2. As this figure shows most of the studied LNCs release about 70% of the loaded drug within 65 minutes with a zero order or Baker-Lonsdale kinetics model indicating the particulate nature of the LNCs. 

Statistical analysis of the release efficiency (RE_65_%) of different formulations of LNCs by Design Expert software shows that increasing amounts of Solutol and Labrafac decreases the RE_65_%, but increasing the cold water volume during dilution step increases the RE_65_%. Lecithin amount has not significant effect on RE_65_%. 

**Figure 3:**
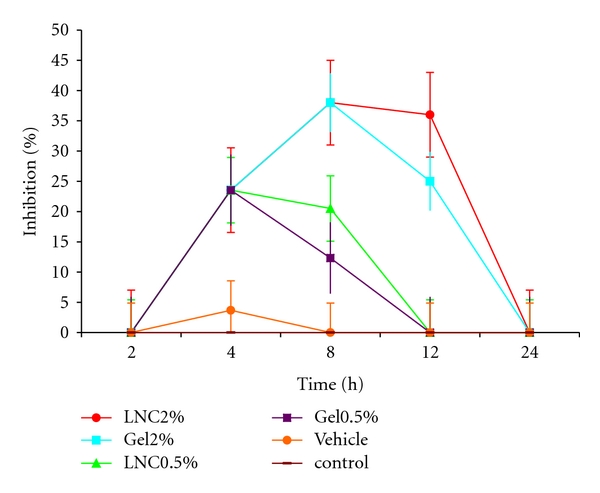
Anti-inflammatory activity (Inhibition %) of ketorolac on paw edema induced with Aerosil injection (0.1 mL of 2.5% w/w) in rats (control), after administration of transdermal gels of optimized ketorolac LNC (0.5% and 2%), the vehicle, and the gel containing 0.5 or 2% of free ketorolac. The formulation of the gels of ketorolac LNC contained (1% Carbomer gel base containing 10% oleic acid and 2% ketorolac loaded in optimized LNCs (S_20_O_25_W_3.5_L_3.25_) prepared by 20% polyethylene glycol hydroxyl stearate, 3.25% lecithin, 25% Labrafac as the oily phase, and cold water as much as 3.5-folds of the total volume of the primary emulsion).

Considering the high solubility of the drug in both water and organic solvents and as there is not any burst effect in the release profiles (Figure 3) it seems that the drug is accommodated in the core of the matrix of LNCs. 

To optimize the gel base for loading LNCs, the Carbomer gels were loaded with the optimized LNC formulation, and permeability of drug through the excised hairless skin of rat was measured. Different formulation of gel bases were designed by an irregular 2-level factorial design and 3 variables each in 2 levels were studied (Table 2). The results of measurements of permeability, flux, and partition coefficient of drug between skin and vehicle are seen in Table 4. 

As shown in Table 4, skin permeation was significantly enhanced by gel of O_10_C_1_. Oleic acid possesses skin penetration enhancing ability. Considering skin structure, water solubility is an important parameter that drastically influences drug permeation profile. The skin is composed of a comparatively lipophilic stratum corneum and hydrophilic viable epidermis and dermis. On the basis of the permeability results, O_10_C_1_ gel containing LNCs of ketorolac showed an optimal balance between lipophilicity and hydrophilicity. This behaviour could be explained by balancing the high percentage of polyethylene glycol hydroxyl stearate (Solutol), the hydrophilic surfactant used in the LNCs, and lipophilic oleic acid used in preparing the gel base. Comparing the results of Table 4 for the optimized gel containing LNCs of ketorolac with the traditional gel of free ketorolac indicates 13 fold increase in permeability for the gel-based LNCs. This indicates a significant increase in permeability and flux of ketorolac when encapsulated in the LNCs indicating a much better affinity of drug to the stratum corneum. On the other hand, Table 4 shows that *K*_*m*_ value for the gel-based LNCs has a much higher value than what is reported for NLC of this drug by Puglia et al. [26]. Generally a high *K*_*m*_ value indicates that the vehicle has a poor affinity for the drug and a low *K*_*m*_ value, indicating a high degree of mutual interaction and the tendency of drug to remain in the vehicle [26]. This shows the LNCs have lower interaction with ketorolac tromethamine than NLCs due to higher hydrophilicity of Solutol used in their formulation. Therefore, the vehicle could not sequester the drug, and the drug is available for diffusion while in other gel formulations this balance is not produced well and skin permeation of the drug is drastically less (Table 4). The optimized gel formulation was considered to contain 1% Carbomer and 10% of oleic acid. This gel was applied for *in vivo* evaluation in controlling the oedema in paw of rat after Aerosil injection. Figure 3 depicts the results of inhibition percent of inflammation after application of free ketorolac 0.5 and 2% in gel base as a conventional gel and the drug encapsulated in LNCs compared to the vehicle and control group of rats.

As this figure shows, conventional and LNC gels containing 0.5% of ketorolac had significant difference (*P* < 0.05) with the control and vehicle group in reducing the edema in paw of rats at all time point of the study except at 24 hr. In these gels the maximum inhibition% in edema happened at 4 h. However, the effect of LNC gels containing 0.5% of ketorolac continued until 8 hr so that the area under the inhibition% time curve (AUC_0–24_) was significantly greater for 0.5% optimized LNC than 0.5% gel (*P* < 0.05) (Table 5). After 12 hr the inhibition% declined for both 0.5% gel and LNC. The anti-inflammatory effect of both 2% LNC and conventional 2% ketorolac gels lasted for more than 12 hr after drug administration. The difference between AUC_0–24_ gels with other groups was significant (*P* < 0.05), and the difference between 2% LNC and 2% gel was also significant (*P* < 0.05) (Table 5). 

This means that their effect lasts more than other treatments. The high activity of the 2% gel could be attributed to the presence of a high amount of oleic acid, a known skin-penetration enhancer in the vehicle of LNCs. However, the sustained activity of the LNC-based gel even at the end of 12 h could be explained by the drug encapsulated within the LNCs, while the fast onset is explained by the free drug in the outer phase of dispersion. Abdel-Mottaleb et al. [31] studies showed that LNCs caused higher permeation-enhancing effect compared to polymeric nanoparticles, while they had similar permeation to solid lipid nanoparticles (SLN). On the other hand, LNCs had the advantage of lower intradermal drug accumulation as well as higher loading efficiency combined with less stability problems compared to SLN.

## 4. Conclusion

Unlike the NLCs of ketorolac reported before that could not enhance transdermal anti-inflammatory effects of this drug, the LNCs reported in this paper showed 13-fold increases in permeability of ketorolac compared to conventional gels. The partition coefficient of drug between stratum corneum and the vehicle was significantly higher than what is reported for NLCs of this drug. The results of inhibition percent of inflammation induced by Aerosil in the paw of the rats also allow to conclude that encapsulation of ketorolac in LNC-based gel can enhance and prolong the anti-inflammatory effects of this drug for more than 12 h.

## Figures and Tables

**Table 1:** Different variables and their levels studied by Taguchi design for production of nano lipid capsules of ketorolac tromethamine.

Variable	Level		
	I	II	III
Surfactant % (S)	20	25	30
Oil phase % (O)	10	17.5	25
Volume of diluting aqueous phase ml (W)	1.2	2.35	3.5
Lecithin % (L)	1.5	2.35	3.25

**Table 2:** Different variables and their levels studied by 2-level factorial design for production of gels of nano lipid capsules of ketorolac tromethamine.

Variable	Level	
	I	II
Enhancer type	Oleic acid (O)	Propylene glycol (P)
Enhancer %	10	15
Carbomer concentration	0.5	1

**Table 3:** Physical properties of nano lipid capsules of ketorolac tromethamine (RE_65_% is released efficiency percent of drug until 65 min of release test) (results are mean ± SD).

Type of nanoparticles	Average size (nm)	Zeta potential (mV)	Drug loading efficiency (%)	RE_65_ (%)
S_25_O_10_W_2.35_L_5_	56.0 ± 24.9	−3.3 ± 0.7	38.6 ± 2.5	44.4 ± 1.7
S_20_O_10_W_1.2_L_1.5_	59.7 ± 11.4	−1.6 ± 0.4	13.5 ± 0.4	40.6 ± 3.4
S_30_O_10_W_3.5_L_3.25_	20.5 ± 0.7	−3.7 ± 0.3	36.2 ± 3.1	43.2 ± 1.5
S_25_O_25_W_1.2_L_3.25_	221.0 ± 42.2	−1.9 ± 0.1	28.9 ± 0.4	25.0 ± 0.2
S_30_O_17.5_W_1.2_L_5_	39.5 ± 8.2	−1.8 ± 0.1	34.0 ± 0.7	24.4 ± 0.0
S_25_O_17.5_W_3.5_L_1.5_	86.8 ± 2.2	−3.9 ± 0.4	23.0 ± 1.5	38.6 ± 0.0
S_30_O_25_W_2.35_L_1.5_	196.2 ± 4.5	−3.3 ± 0.6	29.8 ± 2.5	26.7 ± 0.0
S_20_O_25_W_3.5_L_5_	54.8 ± 4.2	4.0 ± 0.6	26.6 ± 1.2	39.5 ± 0.4
S_20_O_17.5_W_2.35_L_3.25_	60.5 ± 12.8	−4.2 ± 0.4	34.8 ± 6.8	39.1 ± 0.3
Optimized LNCs (S_20_O_25_W_3.5_L_3.25_)	102.7 ± 0.8	−7.8 ± 0.5	31.0 ± 10.6	37.4 ± 1.3

**Table 4:** Permeability of ketorolac tromethamine loaded in optimized nano lipid capsules through hairless rat skins from different gel vehicles compared with free drug loaded in gel of O_10_C_1_ (results are mean ± SD).

Composition of gel vehicle	Permeability × 10^6^ (cm/sec)	Flux (*μ*g/cm^2^sec)	*K*_*m*_
Free drug in O_10_C_1_ gel	0.6 ± 0.03	0.0006	
O_10_C_1_	8.3 ± 0.6	0.009	74.7
O_10_C_0.5_	4.2 ± 0.4	0.005	26. 5
P_10_C_1_	2.1 ± 0.2	0.002	5.3
O_15_C_1_	3.0 ± 0.6	0.003	12.6
P_15_C_1_	2.5 ± 0.4	0.003	7.87
P_10_C_0.5_	2.6 ± 0.1	0.003	6.68
P_15_C_0.5_	1.0 ± 0.1	0.001	0.25
O_15_C_0.5_	3.7 ± 0.2	0.004	24.53

**Table 5:** Area under the curve of inhibition time after administration of ketorolac tromethamine loaded in optimized nano lipid capsules from different gel vehicles after induction of inflammation in rat paws by Aerosil injection (results are mean ± SD).

Treatment groups	AUC_2–24_ (%.h)
LNC 2%	510.59 ± 7.00
gel %2	488.59 ± 4.86
LNC 0.5%	152.59 ± 5.4
gel 0.5%	119.87 ± 5.89
Vehicle	11.01 ± 4.86
No treatment	0.00 ± 0.00
